# Evaluation of permanent alopecia in pediatric medulloblastoma patients treated with proton radiation

**DOI:** 10.1186/s13014-014-0220-8

**Published:** 2014-11-18

**Authors:** Chul Hee Min, Harald Paganetti, Brian A Winey, Judith Adams, Shannon M MacDonald, Nancy J Tarbell, Torunn I Yock

**Affiliations:** Department of Radiation Oncology, Massachusetts General Hospital and Harvard Medical School, Boston, MA USA; Department of Radiation Convergence Engineering, Yonsei University, Wonju, Korea

**Keywords:** Permanent alopecia, Proton therapy, Pediatric medulloblastoma, Monte Carlo simulation

## Abstract

**Background:**

To precisely calculate skin dose and thus to evaluate the relationship between the skin dose and permanent alopecia for pediatric medulloblastoma patients treated with proton beams.

**Methods:**

The dosimetry and alopecia outcomes of 12 children with medulloblastoma (ages 4-15 years) comprise the study cohort. Permanent alopecia was assessed and graded after completion of the entire therapy. Skin threshold doses of permanent alopecia were calculated based on the skin dose from the craniospinal irradiation (CSI) plan using the concept of generalized equivalent uniform dose (gEUD) and accounting for chemotherapy intensity. Monte Carlo simulations were employed to accurately assess uncertainties due to beam range prediction and secondary particles.

**Results:**

Increasing the dose of the CSI field or the dose given by the boost field to the posterior fossa increased total skin dose delivered in that region. It was found that permanent alopecia could be correlated with CSI dose with a threshold of about 21 Gy (relative biological effectiveness, RBE) with high dose chemotherapy and 30 Gy (RBE) with conventional chemotherapy.

**Conclusions:**

Our results based on 12 patients provide a relationship between the skin dose and permanent alopecia for pediatric medulloblastoma patients treated with protons. The alopecia risk as assessed with gEUD could be predicted based on the treatment plan information.

## Background

Medulloblastoma is the most common malignant brain tumor in children and arises in the posterior fossa. Current studies show that combined modality therapies including surgery, chemotherapy and radiation can achieve a cure in approximately 80% of children with standard risk disease and in excess of 60% in children with high risk disease [1]. With increasing long-term survival, there is a growing concern with treatment-related side effects. Permanent alopecia can have a profound impact on a child’s quality of life (QoL), contributing to low self-esteem and adversely affect psychosocial functioning [2,3].

To mitigate the side effects of photon radiation therapy, various radiation delivery methods have been developed with an improved conformity of radiation dose delivery, but these refinements in the photon techniques still entail significant entrance and exit dose. Compared with photon techniques, proton radiation affords a high radiation dose to the target volume while decreasing the amount of normal tissue irradiated by a factor of 2-3 times [4,5] and a few studies have estimated the benefits in decreased second tumor rates and long term costs of caring for children [6]. Ongoing studies document some of the partially mitigated late effects of neurocognitive deficits, hearing deficits, and endocrine deficits [7].

The potential risk of permanent alopecia after photon cranial irradiation in adults has been studied previously [8]. However, little is known about the differences in sensitivity to radiation with regards to alopecia outcomes in children. Due to the fact that many organs and tissues are still developing, children may be more sensitive than adults to radiation. In addition to the normal tissue toxicity and tolerances that adults are subject to, further growth and development is stunted in children [9].

We made the clinical observation that after 36 Gy(RBE) and concurrent chemotherapy, hair regrowth in pediatric patients was often incomplete. A few young patients treated with intensive or concurrent chemotherapy (beyond vincristine) also had some signs of permanent alopecia at doses as low as 23.4 Gy(RBE) of proton radiotherapy. Discussions with clinicians at other centers (not proton specific) have revealed that this is a widespread problem especially with the current Children’s Oncology Group (COG) regimen of daily carboplatin and 36 Gy of CSI in the high risk study.

Permanent alopecia has been a major source of dissatisfaction with our patients which was consistent with a finding by Kinehan et al from the Childhood Cancer survival studies [3]. Permanent alopecia was statistically significantly associated with worse QoL scores for the global status indicator as well as all three subsets of anxiety, somatization and depression [3]. Therefore, the purpose of this study is three fold: 1) to determine the threshold CSI dose causing permanent alopecia in children with medulloblastoma, 2) to identify other contributing factors such as chemotherapy; and 3) to use this data to improve the proton CSI technique to further decrease skin dose and thus decrease the risk of permanent alopecia.

## Materials and methods

### Patient selection and treatment planning

Twelve pediatric patients with medulloblastoma (median age 6, range 4-15 years) were treated with proton CSI and boost at Massachusetts General Hospital (MGH) whose permanent alopecia outcomes were graded according to Common Terminology Criteria for Adverse Events (CTCAE) v4.0 [10]. For this study only the brain fields were considered. CSI (whole brain) doses ranged from 23.4 to 36.0 Gy(RBE). Boost fields consisted of either whole posterior fossa or tumor bed involved field and were treated to a total dose of 54 Gy(RBE) except in case 7 where 50.4 Gy(RBE) was used (Table 1).

**Table 1:** **Patient characteristics**

**Case no.**	**Age (y)**	**Gender**	**Prescribed dose (Gy(RBE))**		**Chemotherapy**	**Risk-group**
			**CSI**	**Boost**		
1	7	F	23.4	30.6	CD	SR
2	4	F	23.4	30.6	CD	SR
3	5	M	23.4	30.6	HD	SR
4	10	M	23.4	30.6	CD	SR
5	4	M	23.4	30.6	HD	SR
6	4	M	27.0	27.0	HD	HR
7	6	M	30.6	19.8	HD	HR
8	15	M	36.0	18.0	CD	HR
9	9	M	36.0	18.0	CD	HR
10	8	F	36.0	18.0	CD	HR
11	5	F	36.0	18.0	CD	HR
12	4	F	36.0	18.0	HD	HR

*Abbreviations:* *M* male, *F* female, *HD* high-dose chemotherapy, *CD* conventional-dose chemotherapy, *SR* standard-risk, and *HR* high-risk.

In order to accurately assess skin dose, the skin was manually defined as the volume from the outer surface of the skin to the depth of about 6-7 mm. If the skin was thinner than 6 mm, the skin was defined from the outer surface of the skull to the outer skin surface. The treatment planning system XiO (Computerized Medical System Inc.) was used for all treatment plans. All patients had completed both chemotherapy and radiation therapy more than 1.25 years before the follow up alopecia grading to ensure adequate time for the regrowth of hair.

### Monte Carlo simulation

The prescribed brain fields have a proton beam range equaling the water-equivalent thickness of the patient’s head. As a range uncertainty safety margin, our institution typically adds an additional 3.5% + 1 mm to the prescribed range. Thus, for medulloblastoma patients, the added range to account range uncertainty of the proton beam in the brain could cause a significant increase in the skin dose. Each treatment plan was re-calculated using a Monte Carlo dose calculation system for two purposes: 1) to avoid potential uncertainties in our analysis due to dose calculation errors, and 2) to better define the actual range uncertainty in these particular cases to determine if the addition 3.5% + 1 mm is truly necessary. This code considers all the modules in the treatment head and the patient geometry by importing patient CT data and simulates primary and secondary particles. Monte Carlo dose calculations are known to be more accurate than a pencil-beam algorithm used in commercial treatment planning systems [11].

Passive scattered proton therapy is associated with a neutron background. Furthermore, there are short-range protons from aperture scattering. Both effects are not modeled in analytical dose calculation algorithms but could contribute to the skin dose. Monte Carlo simulations allow us to consider all secondary particles generated in the treatment head. We calculated the skin dose from secondary particles separately to assess the impact of neutrons, photons, electrons, and secondary protons. Neutrons have a high radiation weighting factor while electrons, photons and secondary protons have a short-range and thus could potentially increase the skin dose at the entrance of the beam into the patient.

### Grading and dose-response analysis

Permanent alopecia was graded with CTCAE v4.0 and analyzed based on the generalized equivalent uniform dose (gEUD) [10,12]. Alopecia grading is as follows: 0 = none; 1 = hair loss of <50% (a different hair style may be required); 2 = hair loss of > =50% (a wig or hair piece is necessary). To assess the skin dose in which the hair follicles are found, the cumulative dose-volume histograms (DVHs) were obtained from the manually contoured skin volume in the XiO treatment planning system. Based on the DVHs of each patient the gEUD was defined as:

1 $$ gEUD=\left(\frac{1}{N}{\displaystyle {\sum}_i^N{D_i}^a}\right){\scriptscriptstyle \frac{1}{a}} $$

Here, D_i_ is the dose in the i’th voxel, N is the number of voxels in the anatomic structure of interest, and ‘a’ is the tumor or normal tissue-specific parameter (we assume a = 10 for skin) that describes the dose-volume effect [13]. To investigate the relationship between the alopecia grade and the skin dose, gEUDs for the CSI fields and boost fields were separately calculated and analyzed. Chemotherapy intensity and its effects on permanent alopecia were also considered.

## Results

### Dose calculation uncertainties when analyzing skin dose

On average, the depth of the scalp follicle is located approximately 4.5 mm from the skin surface in adults and somewhat shallower in younger children, i.e. pediatric scalp skin is somewhat thinner than adult skin. Assuming that the scalp skin is thin and the dose distribution in it is uniform, the average dose of the scalp skin equals the follicle dose.

**Figure 1:**
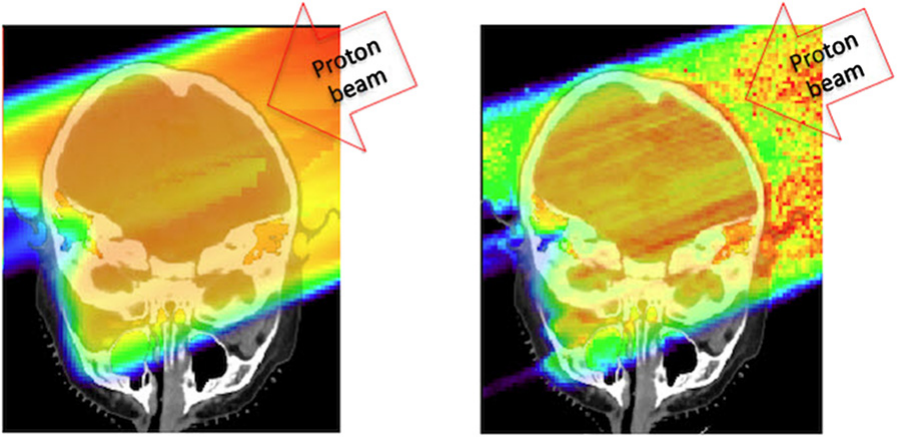
**Proton dose distribution for a single field as calculated with (left) the treatment planning system (XiO) and (right) a Monte Carlo simulation.**

To verify that the range of the proton beam and the following skin dose calculated based on the treatment planning system is correctly predicted, Monte Carlo (MC) simulations were performed. One example is shown in Figure 1 where a beam of 167-mm range was delivered to the patient through the patient-field-specific aperture and compensator. The difference of the average beam range was less than 1.0 mm with root-mean-square-errors of 2.6 mm between XiO and MC simulation resulting in a similar gEUD for the skin dose of 1.73 and 1.70 Gy(RBE), respectively (for 1.8 Gy(RBE) of the prescribed CSI dose). Note that for the comparison purpose only one faction was considered, but the total dose was 54 Gy(RBE). The comparisons between XiO and MC calculations for the other 11 patients treated with field ranges of 151-172 mm were similar.

The proton beams were delivered with a posterior oblique field with a prescription dose as shown in Table 1. The doses include a relative biological effectiveness (RBE) of 1.1, 1 and 10 for protons, electrons and neutrons, respectively. The RBE could potentially be slightly higher in the distal part of the SOBP. This is clinically not considered due to uncertainties in tissue, dose and LET (liner energy transfer) dependency of RBE values. The MC simulated dose distributions are based on primary protons as well as secondary particles generated by proton-induced nuclear interactions in the treatment head. Even though the number of neutrons was about 14% of the primary protons, most of them passed the patient without any interactions because of the low interaction cross-section and thus the skin dose was not appreciably increased. Overall, the skin dose due to all secondary particles was less than 1% of the proton dose.

### Skin dose and grade

**Figure 2:**
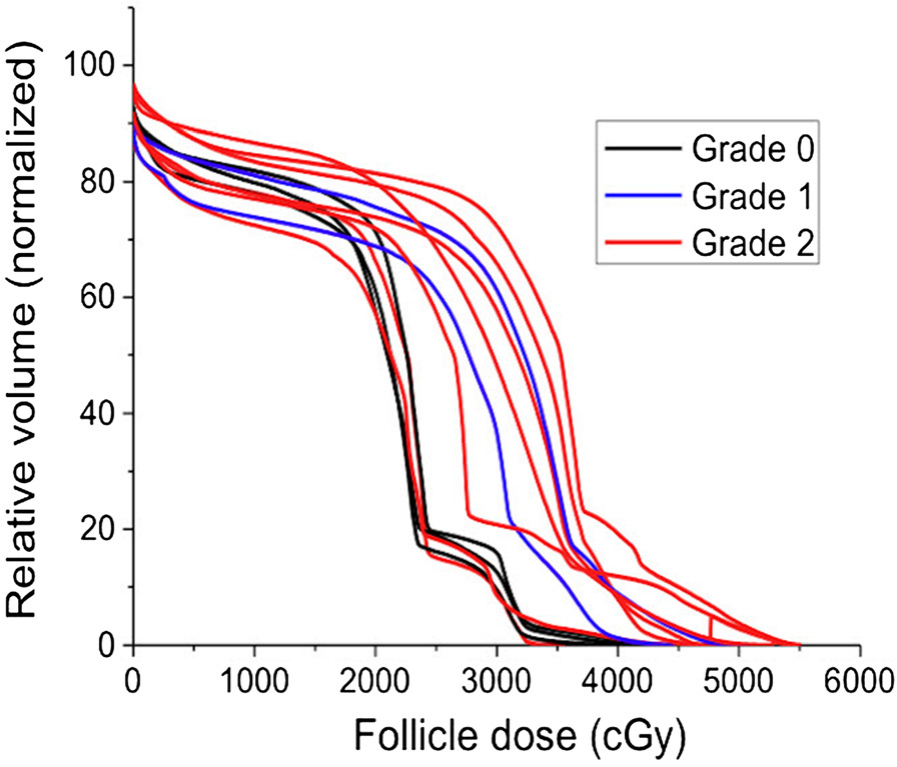
**Dose volume histograms of 12 patients treated with CSI and boost plan along with assigned alopecia grades.**

Despite some patient-to-patient variability, the DVHs of the 12 patients treated with standard CSI and boost treatment show similar patterns (Figure 2). Approximately >70% of the CSI prescription dose was delivered to >50% of the skin volume, and the boost treatment sharply increased the dose to 10-25% of the skin volume. A computed gEUD value for the whole scalp was used and correlated with the CTCAE v4.0 grade.

**Figure 3:**
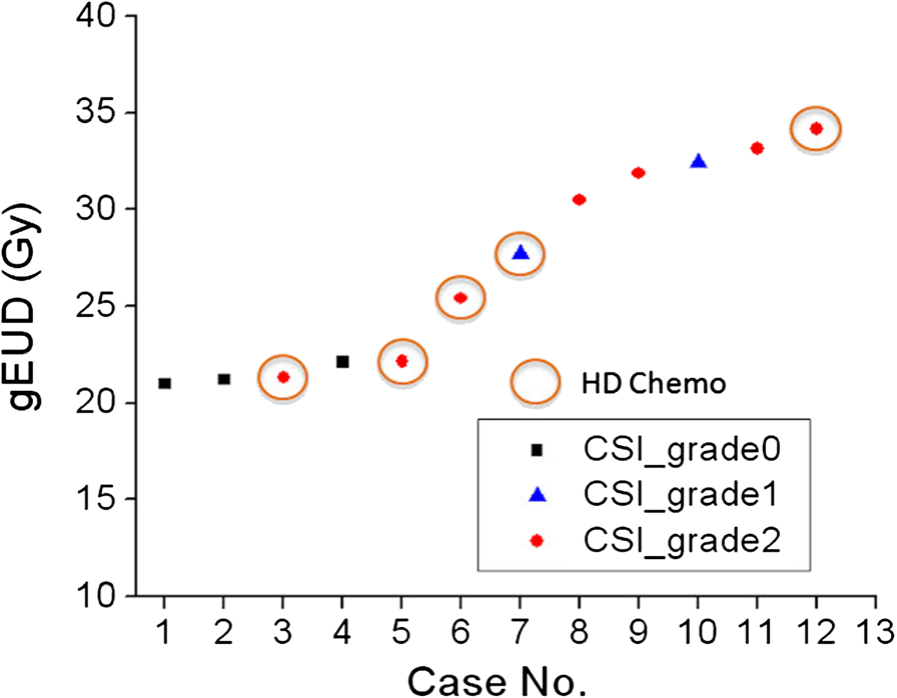
**The gEUD values for 12 patients treated with CSI fields with assigned alopecia grades (boost volume was excluded from the gEUD analysis).** Those patients who also received HD chemotherapy are indicated by circles.

Figure 3 shows the gEUD values with CSI treatment and alopecia grades for the 12 patients. Our results demonstrate that the scalp dose from the CSI field correlates well with alopecia outcomes. However, other factors, such as chemotherapy intensity also play a role. All patients in the current cohort assigned to the high-risk group and treated with > 27.0 Gy(RBE) showed alopecia of grade 1 or 2, while no alopecia was observed with 3 patients (case 1, 2, and 4) out of 5 patients in the standard-risk (lower CSI dose) group. Of the 12 patients, 5 patients (case 3, 5, 6, 7, and 12) were treated with high dose (HD) chemotherapy, and all of them showed permanent alopecia of grade 1 or 2, even though two patients (case 3 and 5) had low skin doses of 21.3 and 22.2 Gy(RBE), respectively. Patients getting lower CSI doses (<23.4 Gy(RBE) seemed to have no alopecia, unless given HD chemotherapy. HD chemotherapy is associated with permanent alopecia in pediatric patients with a skin doses ranges of 21.0 - 34.2 Gy(RBE).

**Figure 4:**
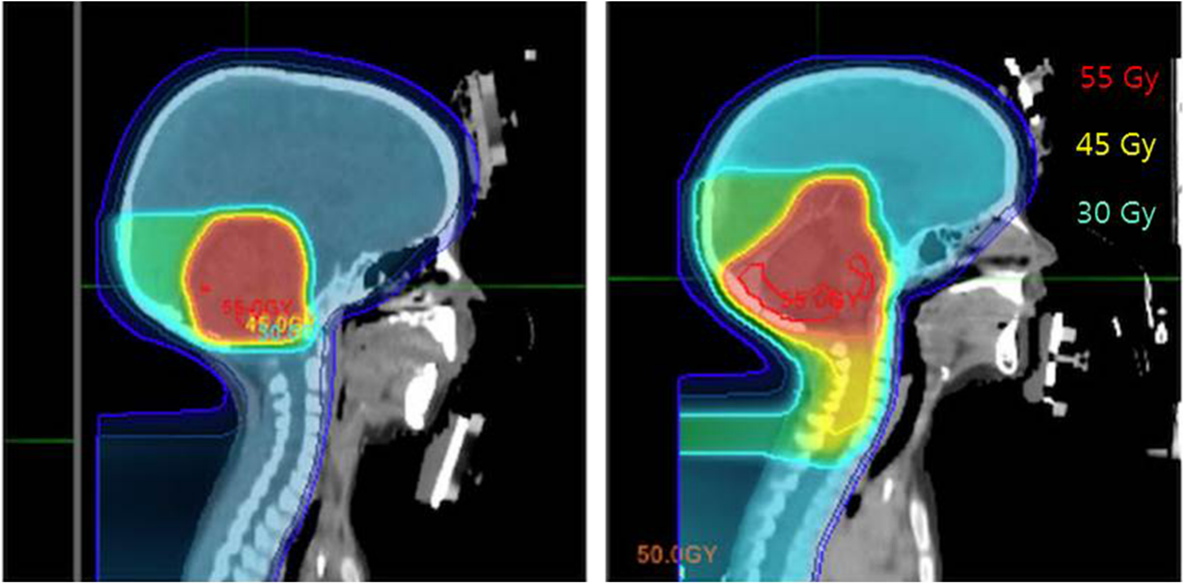
**Proton dose distributions of the patient 5 (left) and 6 (right) treated with CSI and boost field.**

As one might expect, if the skin dose in the posterior fossa region that receives dose from the boost plan is included in the gEUD analysis, the correlation of prescription dose and alopecia is inconsistent. For example, in the cases of patients 5 and 6, both treated with a similar prescribed boost dose of 30.6 and 27.0 Gy(RBE) as shown in Figure 4, respectively, the gEUD values in the skin were about 13.9 and 32.0 Gy(RBE), respectively. Case 5 was treated with an involved field technique, which includes just the primary tumor bed plus a 1.5 cm margin for CTV. However, case 6 includes the whole posterior fossa in the boost. Therefore, the target volume for case 6 was both more superficial and much larger (>3 times) than for case 5. Area dependent grading would be required to consider the boost region separately. Clearly though, the larger and more superficial the posterior fossa boost volume is, the higher the dose to the skin in this region.

**Figure 5:**
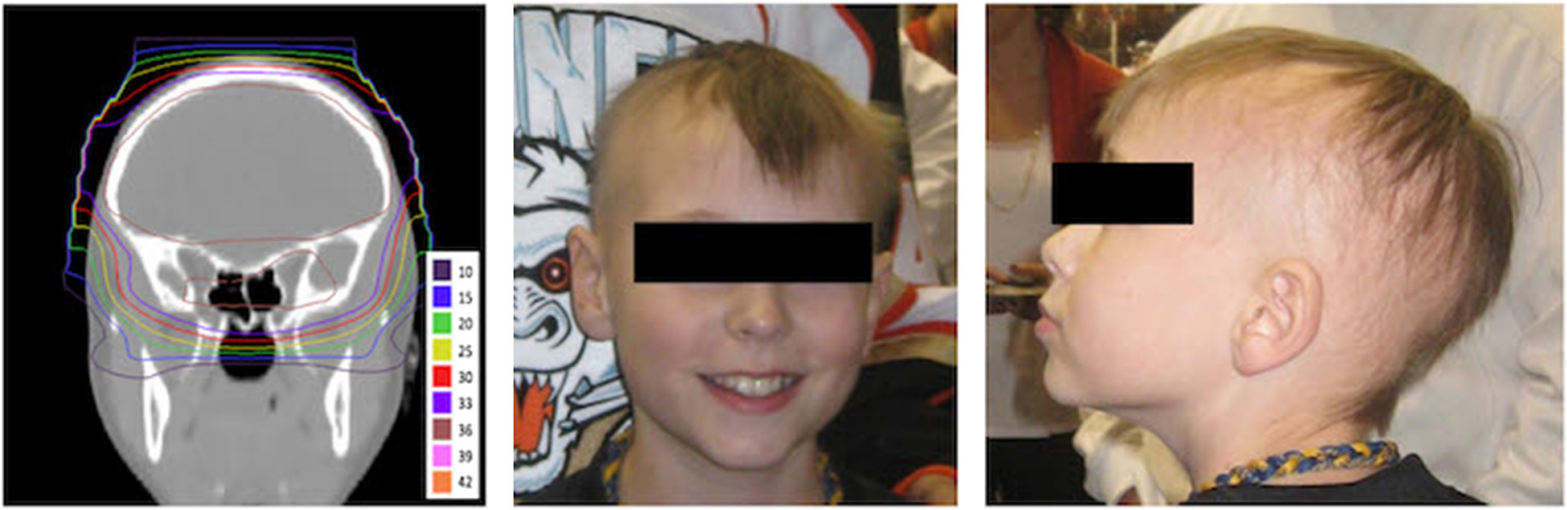
**Planned dose distribution of CSI field (left) with frontal (middle) and left lateral views (right) of Case 8.**

We show, for example, one patient with permanent CTC grade 2 alopecia over most of his scalp sparing only the hair in a strip at the vertex of his head. The vertex hair regrowth was in the area of skin that received 30 Gy(RBE) or less (Figure 5). These findings indicate reduction of range and modulation (using compensators) can reduce skin dose from proton therapy partially mitigating the risk of permanent alopecia.

## Discussion

To understand the dose dependence of radiation-induced alopecia, a numerical method has been used in calculating the local skin dose in the skin depth (e.g. 4-5 mm) at each region of interest [8]. However, our study showed that skin dose could be rationally assessed using gEUD based on DVHs obtained from the treatment planning system for the CTCAE-based alopecia grading. This is a very simple but powerful method in assisting the oncologist, physicist, and dosimetrist to predict potential alopecia risk during treatment planning in the CSI portion of treatment for medulloblastoma.

Based on a gEUD analysis, it was suggested that a mean dose of ~21 Gy(RBE) could be considered as the threshold for permanent alopecia for pediatric patients treated with high dose chemotherapy, but the alopecia threshold dose may be higher, 30 Gy(RBE) with conventional chemotherapy. This relationship excludes the boost dose in the calculation. With the limited number of cases in our study, the results of permanent alopecia in a pediatric population are notably different compared to the adult study correlating radiation dose to permanent alopecia [8]. They estimated that 43.0 Gy to the skin causes permanent alopecia in 50% of adult patients.

Previous studies had demonstrated a clear advantage of proton beams in a dosimetric comparison with conventional X-ray and intensity-modulated photon therapy (IMPT), particularly in the posterior fossa and spinal column in children with medulloblastoma [5,7]. However, for cranial irradiation proton radiation does not show a skin sparing advantage over photons. This is mainly due to the added range uncertainty margin of 3.5% of the prescribed range + 1 mm. This caused the exit skin dose to be as high as ~80% + of the prescribed target dose. Thus, eliminating the 3 mm margin added for the uncertainty of the dose calculation algorithm effectively reduces skin dose and has been undertaken at our institution in the higher CSI dose patients. Permanent alopecia outcomes using this improved technique are still immature as of the date of submission of this manuscript as many patients are still receiving chemotherapy or not sufficiently far from therapy to securely assess permanent alopecia.

The boost delivers additional dose to the skin in the posterior fossa region and it has potential to affect the CTCAE-based alopecia grading as seen for cases 5 and 6. It is required to define the area-dependent alopecia grading to quantitatively determine the effects of the boost fields. The CTCAE-based grading only supports the gross alopecia grading over the scalp skin. Assessing the relationship between the dose in the microscopic area and their alopecia grade may offer the better understanding for the potential of alopecia with the radiation therapy. The volume of the medulloblastoma boost field (tumor bed involved field versus whole posterior fossa) is a study question in the latest COG (Children’s Oncology Group) trial. The study should definitively answer the question whether the larger field is ultimately needed for disease control. However, clearly, the smaller tumor bed involved field boost treats on average less skin (presented above) and less normal brain [14].

## Conclusion

Skin dose could be correlated with permanent alopecia as well as the use of intensive chemotherapy. Our study using Monte Carlo dose calculation show that skin dose could be rationally assessed using gEUD based on DVHs obtained from the treatment planning system for alopecia grading.

## Abbreviations

RBE: Relative biological effectiveness
CSI: Craniospinal irradiation
gEUD: Generalized equivalent uniform dose
QoL: Quality of life
COG: Children’s Oncology Group
CTCAE: Common Terminology Criteria for Adverse Events
DVH: Dose-volume histogram
MC: Monte Carlo
HD: High dose chemotherapy
CD: Conventional dose chemotherapy
IMRT: Intensity-modulated photon therapy
